# Senescence-Driven Inflammatory and Trophic Microenvironment Imprints Mesenchymal Stromal/Stem Cells in Osteoarthritic Patients

**DOI:** 10.3390/biomedicines11071994

**Published:** 2023-07-14

**Authors:** Giuseppe Fusi, Michael Constantinides, Christina Fissoun, Lydiane Pichard, Yves-Marie Pers, Rosanna Ferreira-Lopez, Veronique Pantesco, Christophe Poulet, Olivier Malaise, Dominique De Seny, Jean-Marc Lemaitre, Christian Jorgensen, Jean-Marc Brondello

**Affiliations:** 1 IRMB, University Montpellier, INSERM, 34295 Montpellier, France; giuseppe.fusi@outlook.com (G.F.); michael.constantinides.89@gmail.com (M.C.); christina.fissoun@inserm.fr (C.F.); ym-pers@chu-montpellier.fr (Y.-M.P.); rosannaferreiralopez@gmail.com (R.F.-L.); veronique.pantesco@inserm.fr (V.P.); jean-marc.lemaitre@inserm.fr (J.-M.L.); christian.jorgensen@inserm.fr (C.J.); 2 SAFE-iPSC Facility INGESTEM, Montpellier University Hospital, 34298 Montpellier, France; lydiane.pichard@inserm.fr; 3 Clinical Immunology and Osteoarticular Diseases Therapeutic Unit, Montpellier University Hospital, 34298 Montpellier, France; 4 Laboratory and Service of Rheumatology, GIGA-I3, Université de Liège, 4000 Liege, Belgium; christophe.poulet@chuliege.be (C.P.); olivier.malaise@chuliege.be (O.M.); ddeseny@chuliege.be (D.D.S.)

**Keywords:** senescence, MSC, osteoarthritis

## Abstract

Senescent cells promote progressive tissue degeneration through the establishment of a combined inflammatory and trophic microenvironment. The cellular senescence state has therefore emerged as a central driving mechanism of numerous age-related diseases, including osteoarthritis (OA), the most common rheumatic disease. Senescence hallmarks are detectable in chondrocytes, synoviocytes and sub-chondral bone cells. This study investigates how the senescence-driven microenvironment could impact the cell fate of resident osteoarticular mesenchymal stromal/stem cells (MSCs) that are hence contributing to OA disease progression. For that purpose, we performed a comparative gene expression analysis of MSCs isolated from healthy donors that were in vitro chronically exposed either to interferon-gamma (IFN-γ) or Transforming Growth Factor beta 1 (TGFβ1), two archetypical factors produced by senescent cells. Both treatments reduced MSC self-renewal capacities by upregulating different senescence-driven cycle-dependent kinase inhibitors. Furthermore, a common set of differentially expressed genes was identified in both treated MSCs that was also found enriched in MSCs isolated from OA patients. These findings highlight an imprinting of OA MSCs by the senescent joint microenvironment that changes their matrisome gene expression. Altogether, this research gives new insights into OA etiology and points to new innovative therapeutic opportunities to treat OA patients.

## 1. Introduction

During a human’s lifespan, upon extrinsic or intrinsic cues, cells from numerous tissues, including skeletal joint and bone, accumulate senescence hallmarks such as p16^INK4a^ and p21^WAF1^ [1,2]. These non-proliferative so-called senescent cells produce a specific secretory profile composed of inflammatory, catabolic and trophic factors, also called the senescence-associated phenotype (SASP). When chronically produced, this SASP triggers senescence propagation and tissue degeneration and thus contributes to the onset of numerous age-related pathologies [1,3]. Among them, the one main rheumatic disease is osteoarthritis (OA) [4]. Senescence markers indeed accumulate in OA articular chondrocytes (for review [4]) while OA still remains a pathology with no validated curative treatment [5].

Resident osteochondral progenitors, also called mesenchymal stromal/stem cells (MSCs), are found in joint and bone compartments [6]. These resident MSCs are essential for tissue homeostasis through their differentiation capacities into chondrocytes and osteocytes [6]. Furthermore, following injuries, MSCs orchestrate osteoarticular repair through their migratory, juxtracrine and paracrine immune-modulatory properties to dialogue with other damaged cells within tissues [7]. Nevertheless, the role of these MSCs in OA disease development remain elusive because when isolated from OA patients these stem cells retain their in vitro differentiation properties [8]. Remarkably, it has been recently shown that local injection of senescent MSCs can drive in young healthy mice an OA-like phenotype [9,10]. These results propose that MSCs are also actors in OA etiology through a non-autonomous cell mechanism. 

To determine how changes in MSC functions participate to OA development, we asked whether the inflammatory and trophic secretory profile produced by articular senescent cells could impact their transcriptomic profile. For that purpose, healthy donor MSCs were chronically exposed to such senescence-driven microenvironments in order to evaluate changes in MSC self-renewal and gene expression profiles. Finally, through in silico comparative and gene set enrichment analyses, the common senescence-driven gene signature found in these treated healthy MSCs was sought in the transcriptional profile of MSCs isolated from OA patients.

## 2. Materials and Methods

### 2.1. Human MSCs and In Vitro Treatments

A previously described collection of MSC samples from age-matched healthy donors and patients with OA was obtained from bone marrow aspirates [11,12] upon signature of the informed consent according to the French and European ethics guidelines. Briefly, MSCs were selected by plastic adherence and grown in α-MEM Eagle (Ozyme, Saint-Cyr-l’École, France; Cat#BE12-169F-12) supplemented with 10% fetal bovine serum (Merck, Boston, MA, USA), 1% penicillin/streptomycin and 2 mM L-glutamine. For amplification, cells were seeded at 1000 cells/cm^2^ with a growth medium supplemented with 1 ng/mL human FGF2 (Miltenyi Biotec, Bergisch Gladbach, Germany, Cat# GER 130-104-924). MSC samples from healthy and OA donors were characterized by assessing the expression of cell surface markers by cytometry (CD105+, CD90+, CD73+, CD45- and CD34-) and their capacity to differentiate in vitro into chondrocyte, adipocyte and osteoblastic lines. These analyses confirmed that the MSCs from healthy and OA donors used in this study were proliferative and multipotent stem cells. For senescence induction, healthy human MSCs were incubated with recombinant human TGFβ1 (0.5 ng/mL) (Bio-Techne, Boston, MA, USA, Cat#7754-BH) or recombinant human interferon IFN-γ (40 ng/mL) (Bio-Techne Cat#287-IF) added to the growth medium without FGF2 for 14 days. The medium supplemented with IFN-γ or TGFβ1 was renewed every 2 days.

### 2.2. MSC Self-Renewal Capacities 

The capacity of MSCs to form colonies was assessed by seeding 1500 cells in 35 mm Petri dishes in α-MEM growth medium for 10 days after 14 days of pre-culturing in the presence of IFN-γ or TGFβ1. Colony formation units (CFUs) were counted after Giemsa staining. For each condition, the colony number was set as the mean value of three different donors.

### 2.3. Senescence-Associated β-Galactosidase Activity Quantification 

To detect senescence-associated acidic β-galactosidase (SA-β-Gal)-positive cells, the Senescence Cells Histochemical Staining Kit (Sigma-Aldrich, St. Louis, MI, USA; Cat#CS0030) was used according to the manufacturer’s instructions. Specifically, after 14 days of treatment, 12 × 10^3^ MSCs were plated on 18 mm coverslips for 24 h, fixed with the kit’s fixation solution at room temperature for 8 min and incubated overnight with the SA-β-Gal staining solution (pH = 6.0) to reveal SA-β-Gal activity. Ten images of random fields from each sample were taken by microscopy, and at least 100 cells were counted for each sample. Cells with blue staining in the cytosol were counted as SA-β-Gal-positive using the ImageJ software v1.50b.

### 2.4. Gene Expression Analysis by RT-qPCR 

Total RNA was extracted from cells using the RNeasy^®^ Mini kit (Qiagen; Cat#74106) according to the manufacturer’s instructions. RNA quality and quantity were assessed by spectral analysis (A260/280 nm) using a Nanodrop device and stored at −80 °C until analysis. Then, 500 ng total RNA was reverse transcribed using M-MLV reverse transcriptase (Invitrogen, Waltham, MA, USA; Cat#28025013; 5 U/µL final), a random primer hexamer (Thermo Scientific, Waltham, MA, USA; Cat#S0142; 10 ng/µL final concentration), and dNTP (Roche, Basel, Switzerland; Cat# 1277049; 5 mM final concentration) in M-MLV buffer (Invitrogen; Cat#18057-018) for a total volume reaction of 20 µL. SYBR Green-based quantitative PCR was performed using the LightCycle^®^ 480 SYBR Green I master mix (Roche; Cat#04707516001) and 10 ng of cDNA. All measurements were performed in duplicate for each sample. All qPCR amplifications were run on a LightCycler 480 real-time PCR system (Roche) with the primer sequences shown below.
ARFFor5′CCCTCGTGCTGATGCTACTG3′Rev5′ACCTGGTCTTCTAGGAAGCGG3′*CDKN2B*For5′GACCGGGAATAACCTTCCAT3′Rev5′CACCAGGTCCAGTCAAGGAT3′*CDKN2A*For5′GCTGCCCAACGCACCGAATA3′Rev5′ACCACCAGCGTGTCCAGGAA3′*CDKN1A*For5′ACCGAGGCACTCAGAGGAG-3′Rev5′CAGGTCCACATGGTCTTCCT3′*CDKN1B*For5′CGGCTAACTCTGAGGACACG3′Rev5′CTTCTGAGGCCAGGCTTCTT-3′*CDKN1C*For5′GCGGCGATCAAGAAGCTGT-3′Rev5′GCTTGGCGAAGAAATCGGAGA-3′*RPS9*For5′ATGAAGGACGGGATGTTCAC-3′Rev5′GATTACATCCTGGGCCTGAA-3′

Raw data (Ct values) were analyzed using the comparative Ct method. Gene expression values were calculated relative to the housekeeping gene *RSP9* expression level. The comparative threshold cycle method (ΔΔCT) was used to quantify the relative gene expression, and the obtained data were transformed to exponential 2−ΔΔCT values. The results were compared with the *t*-test and *p*-values < 0.05 were considered significant.

### 2.5. Microarray Analysis of Gene Expression 

To perform RNA analysis with the Affymetrix GeneChip Human Gene 2.1 ST array (Cat#902136), total RNA was extracted from MSC samples using the RNeasy^®^ Mini kit (Qiagen, Hilden, Germany, Cat#52304,) followed by incubation with RNase-Free DNase (Cat#79254 Qiagen) according to the manufacturer’s instructions. Samples were stored at −80 °C until analysis. After RNA quality and quantity assessment by spectral analysis (A260/280 nm) and determination of the RIN number using a BioAnalyzer (Agilent Technologies, Santa Clara, CA, USA, Cat#5067-1511), only samples with RIN >8 were used. CEL file treatments and conversion into CHP format were conducted with GCRMA software on Console v1.4.1. Following gene expression data acquisition, CHP files were analyzed using the Transcriptome Analysis Console (TAC) 4.0 software (Thermo Scientific, Waltham, MA, USA) in order to generate Volcano Plots and a Principal Component Analysis (PCA). The heatmap dendrograms were built using Euclidean distance as the dissimilarity metric and the average linkage for the structure. The gene expression Fold-change filter was set to ±1.3, and the gene expression *p*-value was considered relevant when <0.05. With the small number of MSC donors (*n* = 3), a frequency analysis was performed to be more selective and stringent. Then, the results of each non-treated sample were crossed with those of each treated sample in a donor-independent method. Thereby, nine sets of comparisons were obtained from only three donors. Finally, lists of up- and downregulated genes were obtained for either IFN-γ- or TGFβ1-treated MSC samples compared to untreated controls (with a frequency higher than ≥5/9).

### 2.6. Gene Ontology Analysis, Gene Set Enrichment Analysis (GSEA) and Venn Diagram Representation

GO analyses were obtained using the web-based Gene Set Analysis Toolkit (http://www.webgestalt.org, accessed on 1 January 2019) with default parameters [13]. GSEA analysis was performed using the publicly available software GSEA v.4.0.2 (http://www.broad.mit.edu/gsea, accessed on 1 January 2019). For each array dataset, gene expression levels were converted into a ranked-based gene matrix with the Signal2Noise metric method. Following the GSEA official website instructions, when required, probe sets were collapsed to a single gene expression signal using the Collapse Probe Utility with default settings. Most of the gene sets were downloaded from the Broad Institute website (http://software.broadinstitute.org/gsea/msigdb/, accessed on 1 January 2019), with the exception of the MSC_SI_SIGN gene set that was created by our team using the list of common differentially expressed genes (DEGs) in the IFN-γ- and TGFβ1-treated MSC samples. According to GSEA protocol, gene sets with sizes >500 or <10 were excluded. Phenotype permutation was applied because the number of samples available for each condition was at least equal to 3. The *p*-values for gene sets were computed by permuting the genes 1000 times and were considered relevant if <0.05. The 6-set Venn diagram was designed using the R software v.4.3.1 and the publicly available nVenn algorithm (https://github.com/vqf/nVenn, accessed on 1 January 2019). Affymetrix data obtained in this study are available online: ArrayExpress accession E-MTAB-11669.

### 2.7. Statistical Analysis

All data are presented as median or mean ± SEM. Student’s *t*-test was used for comparisons between experimental groups. For Affymetrix analysis, a one-way analysis of variance (ANOVA) was performed after the eBayes correction method. Data were analyzed using the Prism software v9 (GraphPad Software Inc., La Jolla, CA, USA); *p*-values < 0.05 were considered significant (* *p* < 0.05; ** *p* < 0.01; *** *p* < 0.001; **** *p* < 0.0001).

## 3. Results and Discussion

### 3.1. The Senescence-Driven Microenvironment Mediated by Interferon-Gamma (IFN-γ) Reduces Self-Renewal and Triggers Senescence Features in Healthy Human MSCs

**Figure 1:**
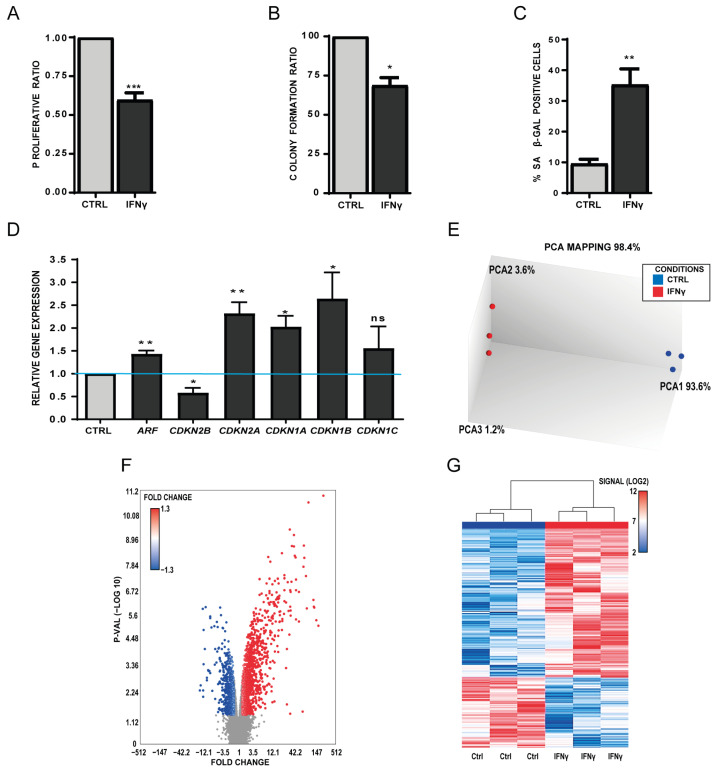
Incubation of human primary MSCs with IFN-γ for 14 days induces senescence features. (**A**) Normalized proliferation ratio of treated and untreated (CTRL) MSCs after 14 days of incubation with IFN-γ (mean ± SEM). (**B**) Colony-forming unit capacities of MSCs after 14 days of incubation with IFN-γ or not (CTRL) (mean ± SEM). (**C**) Expression of senescence-associated β galactosidase (SA-β-gal; blue) in IFN-*γ*-treated and untreated (CTRL) MSCs. The bar chart represents the percentage of SA-β-gal-positive cells (mean ± SEM) in the whole culture (*n* = 3). (**D**) Relative expression of senescence-associated genes in IFN-γ treated and untreated (CTRL) MSCs (set to 1 for all genes), normalized to the expression level of the housekeeping gene RSP9 (mean ± SEM). (**E**) Principal Component Analysis (PCA) of the Affymetrix data for clustering validation of IFN-γ-treated and untreated MSC samples. (**F**) Volcano Plot for IFN-γ-treated MSCs, showing the distribution of significance for all genes. Blue and red dots represent genes with significant relative expression change compared with the untreated samples (*p*-value < 0.05 and |Fold Change| > 1.3). (**G**) Heatmap showing that IFN-γ-treated and untreated MSCs can be differentiated based on their expression profile. Heatmap was constructed using only genes significantly differentially expressed between conditions (*p*-value < 0.05 and |Fold Change| > 1.3). (* *p* < 0.05; ** *p* < 0.01; *** *p* < 0.001). ns: no statistical.

We selected IFN-γ as an archetypical inflammatory cytokine produced by senescent cells [1]. Next, the impact of such a senescence-driven inflammatory microenvironment on MSC self-renewal properties and gene expression profiles was determined. For that, proliferative MSCs isolated from three healthy donors with recombinant IFN-γ (40 ng/mL) were incubated for 14 days. At the end of the incubation period, cell proliferation was significantly decreased in treated MSCs compared with untreated controls (mean ± SEM decrease = −42 ± 0.04%, *p* = 0.0002) (Figure 1A), as well as their self-renewal capacities as indicated by the lower CFU number (mean ± SEM decrease = −32% ± 4.87%; *p* = 0.01) (Figure 1B). Conversely, the percentage of senescent cells hallmarked by the SA-β-Gal-staining was increased in IFN-γ-treated cells (mean ± SEM increase = 35.3% ± 4.38%; *p* = 0.004) (Figure 1C). These data suggest that this chronic treatment triggers senescence only in parts of the MSC population. This assumption was next confirmed by an RT-qPCR analysis of senescence-regulatory cell cycle inhibitors. The results show a significant upregulation of the *CDKN2A*, *CDKN1A*, *CDKN1B*, *CDKN1C*, and *ARF* genes and a slight decrease in the level of *CDKN2B* in IFN-γ-treated MSCs compared with control (Figure 1D). Next, to identify differentially expressed genes (DEGs) between IFN-γ-treated and control MSCs, a genome-wide transcriptomic analysis was performed using the Affymetrix Human Gene ST 2.1 array on total RNAs isolated from three different IFN-γ-treated and untreated MSCs. Principal Component Analysis (PCA) mapping followed by Volcano chart plotting allowed us to show the different gene distribution [−log10(*p*-value)] vs. Fold change in the two groups (Figure 1E,F). The heatmap of DEGs (Fold change < |1.3|, *p* < 0.05) (Figure 1G) showed that treated and control samples could be differentiated based on their expression profiles (1388 upregulated genes and 670 downregulated genes).

### 3.2. The Senescence-Driven Microenvironment Mediated by TGFβ1 Triggers a Senescence-Like Phenotype in Healthy MSCs

**Figure 2:**
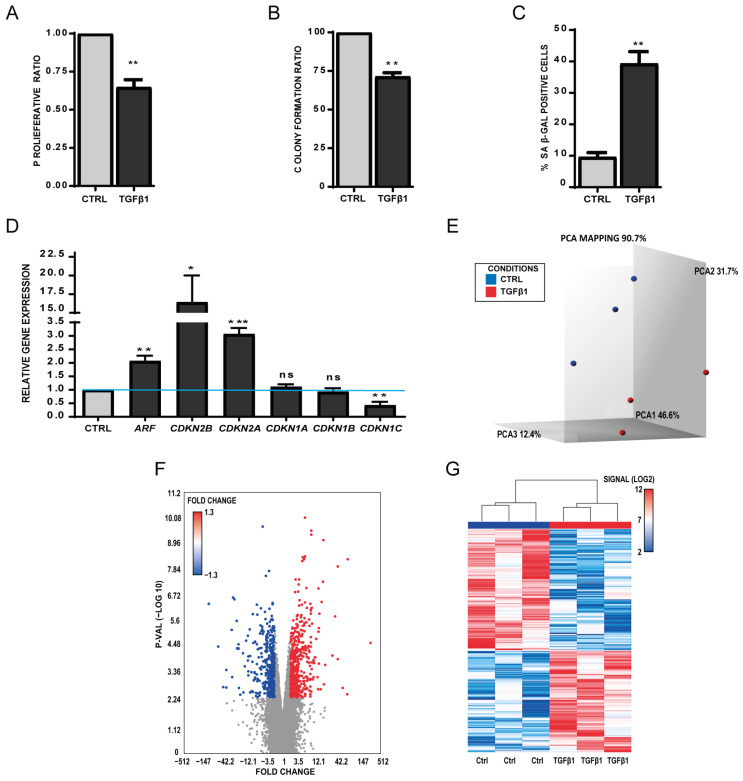
Incubation of human primary MSCs with TGFβ1 for 14 days induces a senescence-like phenotype. (**A**) Normalized proliferation ratio of treated and untreated (CTRL) MSCs after 14 days of incubation with TGFβ1 (mean ± SEM). (**B**) Colony-forming unit capacities of MSCs after or without (CTRL) incubation with TGFβ1 for 14 days (mean ± SEM). (**C**) Expression of senescence-associated β galactosidase (SA-β-gal; blue) in TGFβ1-treated and untreated MSCs. The bar chart represents the percentage of SA-β-gal-positive cells (mean ± SEM) in the whole sample (*n* = 3). (**D**) Relative expression of senescence-associated genes in TGFβ1-treated and untreated (CTRL) MSCs (set to one for all genes), normalized to the housekeeping gene *RSP9* expression level (mean ± SEM). (**E**) Principal Component Analysis (PCA) of Affymetrix data for clustering validation of TGFβ1-treated and untreated MSC samples. Same comments as for Figure 1. (**F**) Volcano Plot showing the distribution of significance for all genes in TGFβ1-treated MSCs compared with control. Dots represent genes with significantly different relative expression (*p*-value < 0.05 and |Fold Change| > 1.3) between conditions. (**G**) Heatmap showing that TGFβ1-treated and untreated MSCs can be clustered based on their expression profiles. Heatmap constructed using only significantly differentially expressed genes (*p*-value < 0.05 and |Fold Change| > 1.3). (* *p* < 0.05; ** *p* < 0.01; *** *p* < 0.001, ns: no statistical).

In addition to inflammatory factors, senescent cells also produce trophic factors, such as TGFβ1, in order to reshape tissue structure [1,2]. Therefore, the same previous MSCs from three healthy donors with recombinant TGFβ1 (0.5 ng/mL) were incubated, and after 14 days of treatment, cell proliferation (mean ± SEM decrease = −31 ± 0.06%, *p* = 0.003) (Figure 2A) and self-renewal capacities (CFUs; mean ± SEM decrease= −29% ± 2.99%; *p* = 0.008) (Figure 2B) were significantly reduced in the treated compared with untreated MSC samples. Moreover, the percentage of SA-β-Gal-positive senescent cells was significantly increased in TGFβ1-treated cells (mean ± SEM increase = 39.3 ± 4.84%; *p* = 0.004) (Figure 2C). The RT-qPCR analysis showed a significant increase in CDKN2B, CDKN2A and ARF mRNA levels, a significant downregulation of CDKN1C, and no change in CDKN1A and B mRNA levels (Figure 2D). Altogether, our data demonstrate that, like for IFN-γ, TGFβ1 induces a senescence-like phenotype in MSCs but with a different cell cycle regulatory gene profile. Next, genome-wide transcriptomic data were obtained by performing Affymetrix analysis for TGFβ1-treated MSCs (*n* = 3/each), which helped determine a list of specific DEGs. PCA mapping and a Volcano chart plotting (Figure 2E,F) showed the different gene distribution [−log10(*p*-value)] vs. Fold change in the two groups. The heatmap of DEGs (Fold change < |1.3|, *p* < 0.05) (Figure 2G) showed that treated and control samples could be differentiated based on their expression profiles (318 upregulated genes and 381 downregulated genes).

### 3.3. Common Gene Set of Both Treated MSCs Is Enriched in MSCs Isolated from OA Patients

**Figure 3:**
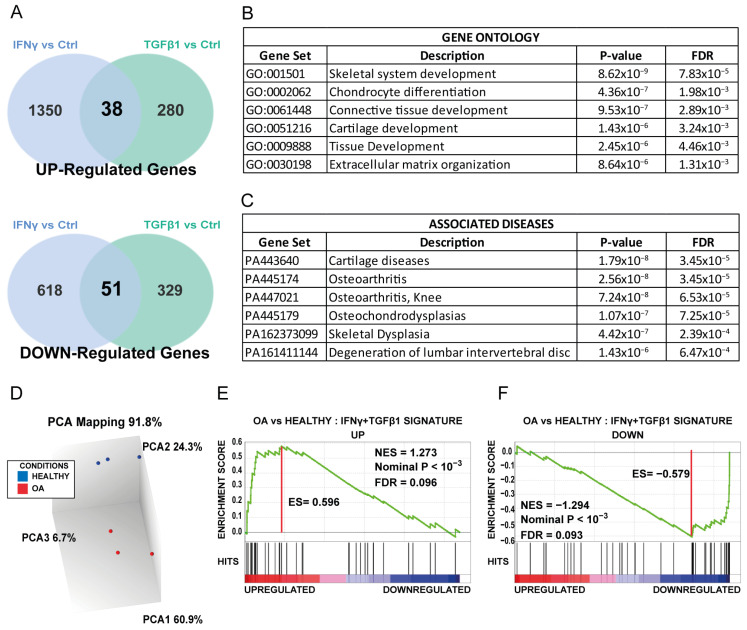
Common gene signature found in IFN-γ- and TGFβ1-treated MSC models is enriched also in MSC from patients with osteoarthritis (OA). MSC exposure to an inflammatory or trophic factor environment regulates the expression of a common set of genes in human primary MSCs. (**A**) Venn diagrams showing the genes that were deregulated in the two in vitro models of senescence induction in MSCs (IFN-γ and TGFβ1) (Affymetrix Gene Chip Analysis). Commonly upregulated (upper) and downregulated (lower) genes are shown. Only significantly deregulated genes (*p*-value < 0.05 and |Fold Change| > 1.3) were included in the analysis. (**B**,**C**) Gene Ontology and Associated Diseases linked to the commonly deregulated set of genes in the MSC models of IFN-γ- and TGFβ1-induced senescence. (**D**) Principal Component Analysis (PCA) of Affymetrix data for clustering validation of OA MSC and healthy MSC samples. (**E**,**F**) Gene set enrichment analysis (GSEA) plots showing significant enrichment of both upregulated (UP) and downregulated (DOWN) genes in the IFN-γ + TGFβ1 signature in OA MSCs compared with MSCs from healthy donors. ES: Enrichment score; NES: normalized enrichment score; FDR: false discovery rate.

Our hypothesis is that both the inflammatory and trophic microenvironments produced by senescent osteoarticular cells in OA disease could alter, in other words, imprint, transcriptionally resident patient MSCs and thus alter their tissue repair and homeostatic properties. To challenge this idea, DEG signatures of MSCs treated with IFN-γ or TGFβ1 were compared. A set of 90 common DEGs, here called “common senescent-driven MSC signature”, was identified (Supplementary Table S1). Among these DEGs, 38 were upregulated and 51 were downregulated in both conditions (Figure 3A). Of note, it was found that ITGA8 (Integrin sub-unit *α*8), which encodes for an important component of the ECM-receptor pathway, was also recently identified as a putative upregulated OA biomarker in an experimental OA rat model [14]. Interestingly, the most downregulated identified gene in this common set encodes for the anti-angiogenic and pro-immunomodulating cytokine Interleukin-12 sub-unit *α* (IL-12a), with so far no link to OA development [15]. To better understand the significance of such a signature, a Gene Ontology (GO) analysis was conducted (Figure 3B). Of note, the results found a significant association with skeletal development (*p* < 0.01). To evaluate whether this core signature can also be linked to rheumatic diseases, a GO analysis was performed using the Associated Diseases datasets. Remarkably, the results highlighted a significant association with OA pathology (Figure 3C). Our findings support the idea that OA disease is the result of combining low-grade inflammation and an altered tissue support microenvironment in the affected joint [5]. Next, the results prompted us to evaluate the presence of that newly identified senescence-driven signature in MSCs, this time isolated from OA patients. To achieve that, the transcriptomic profiles of three OA MSC samples and three MSC samples from age-matched healthy donors were obtained by an Affimetrix analysis. In order to validate the data clustering, PCA mapping was performed for both MSC groups (Figure 3D). Heatmaps and Volcano Plots were also created (Fold change >|1.3|, *p* < 0.05) highlighting the different expression profiles found between OA MSCs and healthy MSCs (Figure S1A). Next, the question of whether the commonly identified senescence-driven MSC signature is also present in the transcriptome of OA MSCs was elucidated using the gene signature enrichment analysis (GSEA) method. The obtained data indicated that the common up and down core signatures were significantly enriched in OA MSCs (Figure 3E,F) (normalized enrichment score (NES) >|1.2|, *p* < 10^−3^). Accordingly, the senescence-driven OA inflammatory and trophic microenvironment clearly imprints the MSC transcriptional profile. This is in accordance with recent preclinical data that have demonstrated that in OA, joint senescence creates a deleterious environment for therapeutic stem cells [16,17].

### 3.4. Identifying Molecular Pathways Altered in OA MSCs by the Senescence-Driven Microenvironment

**Figure 4:**
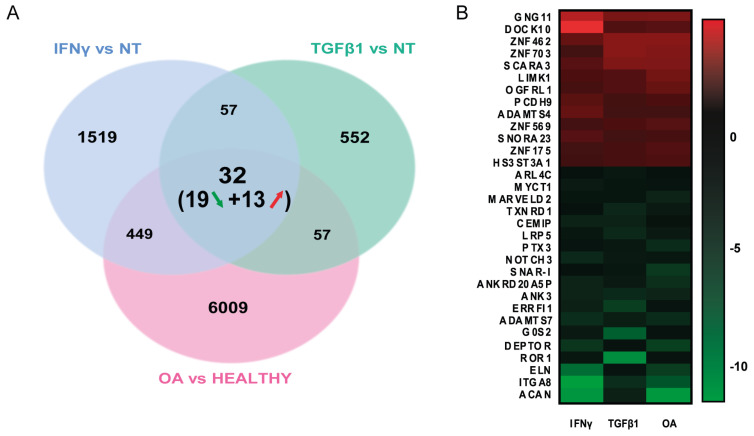

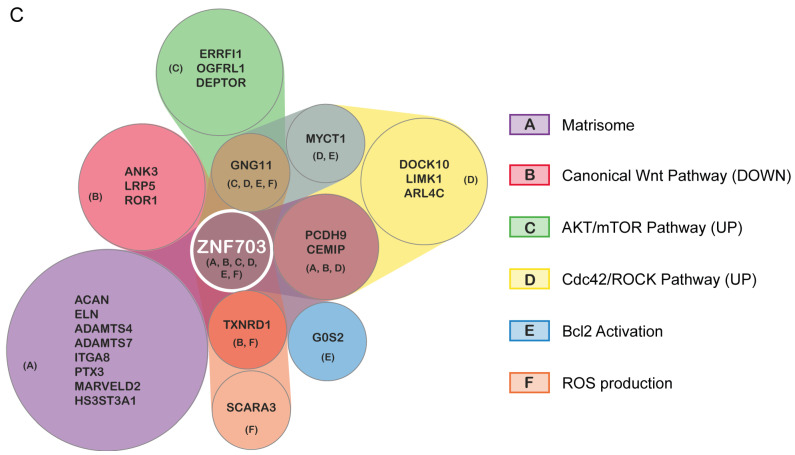
Common genes between OA MSCs and in vitro treated MSC models. (**A**) Venn diagram showing shared upregulated (red arrow) and downregulated (green arrow) genes in IFN-γ-treated MSCs, TGFβ1-treated MSCs and OA MSCs. Only significantly deregulated genes (*p*-value < 0.05 and |Fold Change| > 1.3) compared with controls were included in the analysis. (**B**) Heatmap and list of the 32 commonly deregulated genes from in vitro induced senescent MSC models and OA patient MSCs. (**C**) Six-dimensional pondered Venn diagram showing the main molecular pathways in which the previously listed genes are involved. Overlaps show genes connected to more than one molecular pathway.

To reveal how this novel MSC imprinting can contribute to OA development, a classical gene by gene analysis between OA MSCs and both treated MSCs was performed. The final results show 32 common DEGs, of which 13 were upregulated and 19 were downregulated (Figure 4A,B). An extensive literature-based pathway enrichment analysis for all protein-encoding genes points out that the most enriched molecular pathways concerned genes encoding extracellular matrix (ECM) regulatory and associated proteins (so-called the matrisome) (Figure 4C and Supplementary Table S2). These deregulated genes included several ECM components of cartilage (ACAN, ELN and PTX3), ADAMTS family members (ADAMTS 4 and 7), and also HS3ST3A1, a gene encoding a heparan sulfate-glucosamine 3-sulfotransferase, the most common post-translational modification found on proteo-aminoglycans (PGs) in joints [18] that is altered in OA cartilage [19]. Remarkably, the roles of the matrisome in tissue degeneration/regeneration and stem cell fate decisions are becoming more and more clear [20,21]. ECM matrix components are indeed known to control immune and stem cell fate decisions, for example, during muscle repair (for review [22,23]). Moreover, MSCs are normally recruited at the site of injured cartilage to orchestrate tissue repair and local immune responses [24]. However, here it appears that when exposed to the chronic inflammatory and trophic microenvironment produced by joint senescent cells in OA, these resident MSCs are altered in their ECM gene expression. For instance, the downregulation of ACAN and ELN and the concomitant upregulation of ADAMTS4 (encoding a matrix-degrading enzyme) altogether with an increase in heparan sulfate PG deposition [18] might profoundly modify the nature and the stiffness of the matrisome surrounding MSCs and thus contribute to their defective repair mechanisms during OA progression [21,25]. The second group of genes identified in our core signature was part of the WNT pathway (reviewed in [26]). Downregulated genes such as the WNT receptor components (ROR1/LRP5), their transcriptional target genes (MYCT1, DEPTOR and TXNRD) together with CEMIP, and integrin sub-unit A8 (ITGA8), might also affect MSC motility and recruitment of MSCs to the damaged area [21,27,28]. Finally, the results suggest a central role of the transcription factor ZNF703 (Zinc Finger Protein 703) in the orchestration of all the cellular and metabolic alterations previously discussed. Currently, very little is known about the role of ZNF703 in relation to cartilage development or its homeostasis. Even less is known regarding OA disease, and it should be further studied.

## 4. Conclusions

In this study, our data propose that MSCs isolated from OA patients are transcriptionally affected by the senescence-driven OA inflammatory and trophic microenvironment. This imprinting mainly impacts their capacities to express the adequate ECM/matrisome-encoding genes required for joint homeostasis and tissue repair, hence participating in OA progression. Further experiments should challenge these findings in order to offer innovative strategies to treat patients suffering from OA.

## Data Availability

These data can be found at Array Express accession E-MTAB-11669.

## Supplementary Materials

The following supporting information can be downloaded at: https://www.mdpi.com/article/10.3390/biomedicines11071994/s1, Figure S1: Differential gene expression in MSC from patients with Osteoarthritis (OA) and MSC from healthy donors; Table S1: List of common differentially expressed genes between both IFN-γ and TGFβ1 MSC senescent models.; Table S2: Analytical clustering of common regulated genes between OA and in vitro induced-senescence MSC.
