# Supplementary material for: Senescence-Driven Inflammatory and Trophic Microenvironment Imprints Mesenchymal Stromal/Stem Cells in Osteoarthritic Patients
Source: Biomedicines. 2023 Jul 14;11(7):1994. doi: 10.3390/biomedicines11071994 (PMC10377055; doi:10.3390/biomedicines11071994)
Supplement: Supplementary file 1 [file biomedicines-11-01994-s001.zip › biomedicines-2493313-supplementary.pdf]

Brief Report

Senescence-Driven Inflammatory and Trophic Microenvironment Imprints Mesenchymal Stromal/Stem Cells in Osteoarthritic Patients

Giuseppe Fusi [1], Michael Constantinides [1], Christina Fissoun [1], Lydiane Pichard [2], Yves-Marie Pers [1,3], Rosanna Ferreira-Lopez [1,3], Veronique Pantesco [1], Christophe Poulet [4], Olivier Malaise [4], Dominique De Seny [4], Jean-Marc Lemaitre [1,2], Christian Jorgensen [1,3] and Jean-Marc Brondello [1,*]

[1] IRMB, University Montpellier, INSERM, 34295 Montpellier, France; giuseppe.fusi@outlook.com (G.F.); michael.constantinides.89@gmail.com (M.C.); christina.fissoun@inserm.fr (C.F.); ym-pers@chu-montpellier.fr (Y.-M.P.); rosannaferreiralopez@gmail.com (R.F.-L.); veronique.pantesco@inserm.fr (V.P.); jean-marc.lemaitre@inserm.fr (J.-M.L.); christian.jorgensen@inserm.fr (C.J.)

[2] SAFE-iPSC Facility INGESTEM, Montpellier University Hospital, 34298 Montpellier, France; lydiane.pichard@inserm.fr

[3] Clinical Immunology and Osteoarticular Diseases Therapeutic Unit, Montpellier University Hospital, 34298 Montpellier, France

[4] Laboratory and Service of Rheumatology, GIGA-I3, Université de Liège, 4000 Liege, Belgium; christophe.poulet@chuliege.be (C.P.); olivier.malaise@chuliege.be (O.M.); ddeseny@chuliege.be (D.D.S.)

* Correspondence: jean-marc.brondello@inserm.fr; Tel.: +33(0)4-67-33-57-19

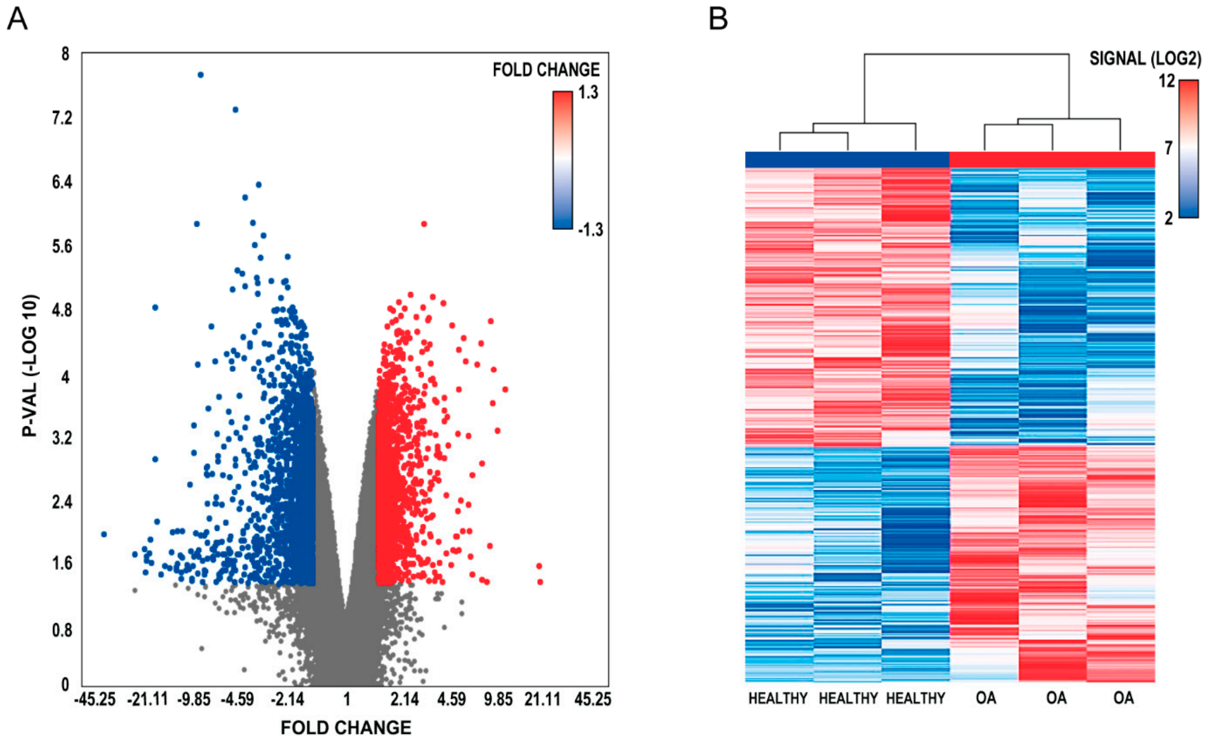

Figure S1. Differential gene expression in MSC from patients with Osteoarthritis (OA) and MSC from healthy donors. (A) Volcano plot for MSC isolated from patients with OA, showing the distribution of significance for all genes. Blue and red dots represent genes with significantly different relative expression (p-value < 0.05 and |Fold Change| > 1.3) compared with healthy

donors. (B) Heatmap showing that MSC isolated from patients with OA and from healthy donors can be differentiated based on the gene expression profile. Heatmap constructed using only significantly differentially genes (p-value < 0.05 and ∣Fold Change∣ > 1.3). Blue and red dots represent genes with significant relative expression changes (p-value < 0.05 and ∣Fold Change∣ > 1.3) in OA MSC compared with healthy MSC.

Supplementary Table S1. List of common differentially expressed genes between both IFN-γ and TGFβ1 MSC senescent models. (Probes bound to uncharacterized encoding genes were excluded).

	**IFN-γ vs NT**		**TGFβ1 vs NT**	
**Gene Symbol**	**Fold Change**	**p-value**	**Fold Change**	**p-value**
ROR1	1,7	0,0014	2,17	0,0004
G0S2	1,89	0,0002	1,48	0,0042
LOC100287497; LOC101926980	1,57	0,0082	1,57	0,0091
ERRFI1	2,18	0,0011	1,65	0,016
LOC105376694	1,73	0,0284	1,58	0,0481
FAM129A	1,67	0,0213	1,39	0,0333
AKR1C3	1,32	0,0138	1,34	0,019
ADIRF	2,15	0,0016	2,47	0,0005
AGAP11; BMS1P3; ADIRF	1,4	0,0051	1,37	0,0013
ITGA8	11,49	0,0042	7,81	0,043
ANK3	2,29	0,0002	3,62	0,0001
LRP5	1,7	0,0046	1,33	0,0283
DYNC2H1	1,73	0,0124	1,48	0,025
AHNAK	1,67	0,0082	1,44	0,0121
CD248	1,4	0,015	2,18	0,0308
TXNRD1	1,37	0,0238	1,7	9,68E-05
APPL2	1,99	0,0001	1,52	0,0114
CEMIP	2,22	0,0053	1,78	0,0368
ACAN	8,5	6,34E-05	16,4	0,0028
MNS1	1,72	0,0005	1,5	0,0025
ADAMTS7	2,75	0,0003	1,85	0,002
FBXW10	1,45	0,046	1,49	0,0291
ANKRD20A5P	2,16	0,001	1,76	0,0161
ANKRD20A5P	2,25	0,0037	2,44	0,0424
MIR3975	2,33	0,0113	1,87	0,0014
NOTCH3	2,57	0,0005	1,31	0,0259
SOCS5	1,53	0,0104	1,79	0,002
ARL4C	1,36	0,0271	2,2	0,0076
MCTS2P	1,44	0,0018	1,43	0,0029
COL6A1	1,56	0,0006	1,33	0,0052
PTPRG	2,16	0,0218	1,6	0,0064
CSTA	1,82	0,0005	1,61	0,0016
PDIA5	1,47	0,0199	1,75	0,0009
PTX3	1,58	0,0028	5,33	0,0007
SNAR-I	1,38	0,0408	2,74	0,0083
TBL1XR1	1,31	0,0065	1,31	0,0203
LOC220729	1,39	0,0003	1,54	0,0379
PDE5A	2,11	0,0003	2	0,0011
MARVELD2	1,65	0,0004	1,91	0,0011
MYCT1	1,94	0,0147	2,1	0,0042
SNX9	1,62	0,0077	2,43	0,0004
TUBE1	1,4	0,0287	1,34	0,0232

MTURN	1,32	0,0291	1,4	0,0329
ELN	6,04	2,73E-06	2,04	0,0003
DPYSL2	1,41	0,0109	1,43	0,0069
DEPTOR	3,13	9,10E-07	1,97	3,08E-05
COL14A1	9,09	9,04E-06	3,25	0,0006
PNMA2	1,3	0,0403	1,87	3,64E-05
PABPC1	1,64	0,0098	1,58	0,0008
EIF3E	1,33	0,0318	1,31	0,0293
CNTNAP3B	1,39	0,0198	1,42	0,0216
LOC100287497; LOC101926980	1,77	0,0199	1,98	0,0138
EPHB2	-2,15	0,0003	-1,33	0,032
PALMD	-1,74	0,0006	-1,42	0,0293
ADAMTS4	-1,91	0,0094	-1,47	0,0024
SNORA23	-1,62	0,0413	-2,55	0,0382
MIR3160-2	-1,48	0,0066	-1,33	0,0173
ACP2	-1,4	0,0181	-1,46	0,0117
CHST11	-1,46	0,0051	-1,51	0,0012
FKBP11	-1,39	0,0136	-1,35	0,0079
PCDH9	-1,74	0,0126	-1,61	0,0092
PLEK2	-1,37	0,0217	-1,37	0,0043
FRMD5	-2,14	0,0121	-1,34	0,0443
LCTL	-1,96	0,018	-1,33	0,0073
MT1CP	-1,7	0,014	-1,69	0,0221
HS3ST3A1	-1,31	0,0261	-2,56	0,0006
HOXB2	-1,79	0,0013	-1,37	0,0144
PLK5	-1,31	0,0373	-1,3	0,0081
ZNF175	-1,32	0,0317	-1,37	0,0251
TSEN34	-1,42	0,0022	-1,34	0,0027
ZNF569	-1,35	0,0052	-1,42	0,0292
NRP2	-2,37	6,11E-05	-1,66	0,0072
IGKV1-39; IGKC	-1,37	0,0352	-1,63	0,0314
ANKRD44	-1,55	0,0021	-1,34	0,0161
DOCK10	-4,5	2,84E-05	-2,41	0,0189
TGM2	-1,78	0,0002	-1,69	0,0024
MRAS	-1,31	0,0249	-1,34	0,006
IL12A	-2,68	0,013	-6,64	0,0295
APBB2	-1,62	0,0205	-1,32	0,0154
SMIM3	-1,4	0,0094	-1,36	0,0279
OGFRL1	-1,4	0,0418	-2,25	0,0004
DCBLD1	-2	0,0146	-1,41	0,0075
LIMK1	-1,47	0,0024	-1,58	0,0092
GNG11	-3,38	0,0027	-2,81	0,0057
TCAF2	-1,34	0,042	-1,37	0,0117
SCARA3	-1,67	0,0066	-1,31	0,0266
ZNF703	-1,33	0,0065	-1,42	0,034
SGK223	-1,68	0,0001	-1,56	0,0016
CLU; MIR6843	-3,05	0,0038	-1,75	0,0267
ZNF462	-1,89	3,07E-05	-1,32	0,0302

Supplementary Table S2. Analytical clustering of common regulated genes between OA and in vitro induced-senescence MSC.

	Gene Symbol	Matrisome	Canonical Wnt Pathway (DOWN)	AKT/mTOR Pathway (UP)	Cdc42/ROCK Pathway (UP)	Bcl2 activation	ROS production	References
UP	ADAMTS4	X						(1)
	DOCK10				X			(3), (4)
	GNG11			X	X	X	X	(5)
	HS3ST3A1	X						(6), (7), (8)
	LIMK1				X			(9)
	OGFRL1			X				(10)
	PCDH9	X	X		X			(11), (12), (13)
	SCARA3						X	(14)
	ZNF703	X	X	X	X	X	X	(15), (16),(17), (18)
DOWN	ACAN	X						(2)
	ADAMTS7	X						(19)
	ANK3		X					(20)
	ARL4C				X			(21)
	CEMIP	X	X		X			(9), (22)
	DEPTOR			X				(23), (24)
	ELN	X						(2)
	ERRFI1			X				(25)
	G0S2					X		(26), (27), (28)
	ITGA8	X						(29)
	LRP5		X					(30)
	MARVELD2	X						(31)
	MYCT1				X	X		(32), (33)
	PTX3	X						(34)
	ROR1		X					(35)
	TXNRD1		X				X	(36), (37), (38)

OA: Osteoarthritis; MSC: Mesenchymal Stem Cells.

(1)	Naito S, Shiomi T, Okada A, et al. Expression of ADAMTS4 (aggrecanase-1) in human osteoarthritic cartilage. Pathol Int. 2007;57(11):703-711. doi:10.1111/j.1440-1827.2007.02167.x
(2)	De Bari C, Dell'Accio F, Tylzanowski P and Luyten FP: Multipotent mesenchymal stem cells from adult human synovial membrane. Arthritis & Rheumatism 44: 1928–1942, 2001.
(3)	Crossland H, Atherton PJ, Strömberg A, Gustafsson T and Timmons JA: A reverse genetics cell-based evaluation of genes linked to healthy human tissue age. FASEB j 31: 96–108, 2017.
(4)	Ruiz-Lafuente N, Minguela A, Muro M and Parrado A: The role of DOCK10 in the regulation of the transcriptome and aging. Heliyon 5: e01391, 2019.
(5)	Slorach EM, Chou J and Werb Z: Zeppo1 is a novel metastasis promoter that represses E-cadherin expression and regulates p120-catenin isoform expression and localization. Genes Dev 25: 471–484, 2011.

(6)	Durak O, de Anda FC, Singh KK, Leussis MP, Petryshen TL, Sklar P and Tsai L-H: Ankyrin-G regulates neurogenesis and Wnt signaling by altering the subcellular localization of β-catenin. Mol Psychiatry 20: 388–397, 2015.
(7)	Hossain MN, Sakemura R, Fujii M and Ayusawa D: G-protein γ subunit GNG11 strongly regulates cellular senescence. Biochemical and Biophysical Research Communications 351: 645–650, 2006.
(8)	Fu S, Fu Y, Chen F, Hu Y, Quan B and Zhang J: Overexpression of MYCT1 Inhibits Proliferation and Induces Apoptosis in Human Acute Myeloid Leukemia HL-60 and KG-1a Cells in vitro and in vivo. Front Pharmacol 9: 1045, 2018.
(9)	Lietman C, Wu B, Lechner S, et al.: Inhibition of Wnt/β-catenin signaling ameliorates osteoarthritis in a murine model of experimental osteoarthritis. JCI Insight 3: e96308, 2018.
(10)	Williams-Pritchard G, Headrick JP, Peart JN. Myocardial Opioid Receptors in Conditioning and Cytoprotection. Pharmaceuticals. 2011; 4(3):470-484. https://doi.org/10.3390/ph4030470
(11)	Agrawal P, Reynolds J, Chew S, Lamba DA and Hughes RE: DEPTOR Is a Stemness Factor That Regulates Pluripotency of Embryonic Stem Cells. Journal of Biological Chemistry 289: 31818–31826, 2014.
(12)	Lyashenko N, Weissenböck M, Sharir A, Erben RG, Minami Y and Hartmann C: Mice lacking the orphan receptor ror1 have distinct skeletal abnormalities and are growth retarded. Dev Dyn 239: 2266–2277, 2010.
(13)	Chen J and Long F: mTOR signaling in skeletal development and disease. Bone Res 6: 1, 2018.
(14)	Luchetti MM, Piccinini G, Mantovani A, et al.: Expression and production of the long pentraxin PTX3 in rheumatoid arthritis (RA). Clinical and Experimental Immunology 119: 196–202, 2002.
(15)	Matsumoto S, Fujii S and Kikuchi A: Arl4c is a key regulator of tubulogenesis and tumourigenesis as a target gene of Wnt–β-catenin and growth factor–Ras signalling. J Biochem 161: 27–35, 2017.
(16)	Korpetinou A, Skandalis SS, Labropoulou VT, Smirlaki G, Noulas A, Karamanos NK and Theocharis AD: Serglycin: At the Crossroad of Inflammation and Malignancy. Front Oncol 3, 2014.
(17)	Yang X, Lu X, Lombès M, et al.: The G(0)/G(1) switch gene 2 regulates adipose lipolysis through association with adipose triglyceride lipase. Cell Metab 11: 194–205, 2010.
(18)	Heckmann BL, Zhang X, Xie X and Liu J: The G0/G1 switch gene 2 (G0S2): Regulating metabolism and beyond. Biochimica et Biophysica Acta (BBA) - Molecular and Cell Biology of Lipids 1831: 276–281, 2013.
(19)	Liu CJ, Kong W, Ilalov K, et al. ADAMTS-7: a metalloproteinase that directly binds to and degrades cartilage oligomeric matrix protein. FASEB J. 2006;20(7):988-990. doi:10.1096/fj.05-3877fje
(17)	Chanalaris A, Clarke H, Guimond SE, Vincent TL, Turnbull JE and Troeberg L: Heparan Sulfate Proteoglycan Synthesis Is Dysregulated in Human Osteoarthritic Cartilage. The American Journal of Pathology 189: 632–647, 2019.
(18)	Welch C, Santra MK, El-Assaad W, et al.: Identification of a Protein, G0S2, That Lacks Bcl-2 Homology Domains and Interacts with and Antagonizes Bcl-2. Cancer Res 69: 6782–6789, 2009.
(19)	Kasper G, Mao L, Geissler S, et al.: Insights into Mesenchymal Stem Cell Aging: Involvement of Antioxidant Defense and Actin Cytoskeleton. Stem Cells 27: 1288–1297, 2009.
(20)	Wang C, Tao B, Li S, et al.: Characterizing the Role of PCDH9 in the Regulation of Glioma Cell Apoptosis and Invasion. J Mol Neurosci 52: 250–260, 2014.
(21)	Lu M, Munger JS, Steadele M, Busald C, Tellier M and Schnapp LM: Integrin α8β1 mediates adhesion to LAP-TGFβ1. Journal of Cell Science 115: 4641–4648, 2002.
(22)	Muri J, Heer S, Matsushita M, et al.: The thioredoxin-1 system is essential for fueling DNA synthesis during T-cell metabolic reprogramming and proliferation. Nat Commun 9: 1851, 2018.
(23)	Ikenouchi J, Furuse M, Furuse K, Sasaki H, Tsukita S and Tsukita S: Tricellulin constitutes a novel barrier at tricellular contacts of epithelial cells. Journal of Cell Biology 171: 939–945, 2005.
(24)	Niklason LE: Understanding the Extracellular Matrix to Enhance Stem Cell-Based Tissue Regeneration. Cell Stem Cell 22: 302–305, 2018.
(25)	Qu S-Y, Sun Y-Y, Li Y-H, Xu Z-M and Fu W-N: YY1 directly suppresses MYCT1 leading to laryngeal tumorigenesis and progress. Cancer Medicine 6: 1389–1398, 2017.
(26)	Kumar A, Chalamalasetty RB, Kennedy MW, Thomas S, Inala SN, Garriock RJ and Yamaguchi TP: Zfp703 Is a Wnt/β-Catenin Feedback Suppressor Targeting the β-Catenin/Tcf1 Complex. Mol Cell Biol 36: 1793–1802, 2016.

(27)	Deroyer C, Charlier E, Neuville S, et al.: CEMIP (KIAA1199) induces a fibrosis-like process in osteoarthritic chondrocytes. Cell Death Dis 10: 103, 2019.
(28)	Kumar A, Chalamalasetty RB, Kennedy MW, Thomas S, Inala SN, Garriock RJ and Yamaguchi TP: Zfp703 Is a Wnt/β-Catenin Feedback Suppressor Targeting the β-Catenin/Tcf1 Complex. Mol Cell Biol 36: 1793–1802, 2016.
(29)	Han H: CSR, a scavenger receptor-like protein with a protective role against cellular damage causedby UV irradiation and oxidative stress. Human Molecular Genetics 7: 1039–1046, 1998.
(30)	Chen L, Shi K, Andersen TL, Qiu W and Kassem M: KIAA1199 is a secreted molecule that enhances osteoblastic stem cell migration and recruitment. Cell Death Dis 10: 126, 2019.
(31)	Cairns J, Fridley BL, Jenkins GD, Zhuang Y, Yu J and Wang L: Differential roles of ERRFI1 in EGFR and AKT pathway regulation affect cancer proliferation. EMBO Rep 19, 2018.
(32)	Gadea G and Blangy A: Dock-family exchange factors in cell migration and disease. European Journal of Cell Biology 93: 466–477, 2014.
(33)	Liang H, Gao K, Zhang Z, Jia R, Li J and Wang C: Functional mechanism of zinc finger protein 703 in in vitro esophageal carcinoma cell line. 7.
(34)	Dai B, Yoo S-Y, Bartholomeusz G, et al.: KEAP1-Dependent Synthetic Lethality Induced by AKT and TXNRD1 Inhibitors in Lung Cancer. Cancer Res 73: 5532–5543, 2013.
(35)	Mah KM and Weiner JA: Regulation of Wnt signaling by protocadherins. Seminars in Cell & Developmental Biology 69: 158–171, 2017.
